# On the shape and structure of the murine pulmonary heart valve

**DOI:** 10.1038/s41598-021-93513-0

**Published:** 2021-07-07

**Authors:** Yifei Liu, Xinzeng Feng, Hao Liu, David W. McComb, Christopher K. Breuer, Michael S. Sacks

**Affiliations:** 1 Center for Electron Microscopy and Analysis, The Ohio State University, Columbus, OH 43210 USA; 2 Department of Materials Science and Engineering, The Ohio State University, Columbus, OH 43210 USA; 3 Willerson Center, Oden Institute for Computational Engineering and Sciences, The University of Texas At Austin, Austin, TX 78712 USA; 4 Department of Biomedical Engineering, The University of Texas At Austin, Austin, TX 78712 USA; 5 Center for Regenerative Medicine, Abigail Wexner Research Institute, Nationwide Children’s Hospital, Columbus, OH 43205 USA; 6 Department of Pediatric Surgery, Nationwide Children’s Hospital, Columbus, OH 43205 USA

**Keywords:** Biological techniques, Imaging, 3-D reconstruction, X-ray tomography, Materials science, Biomaterials, Tissues, Computational biology and bioinformatics, Image processing

## Abstract

Murine animal models are an established standard in translational research and provides a potential platform for studying heart valve disease. To date, studies on heart valve disease using murine models have been hindered by a lack of appropriate methodologies due to their small scale. In the present study, we developed a multi-scale, imaging-based approach to extract the functional structure and geometry for the murine heart valve. We chose the pulmonary valve (PV) to study, due to its importance in congenital heart valve disease. Excised pulmonary outflow tracts from eleven 1-year old C57BL/6J mice were fixed at 10, 20, and 30 mmHg to simulate physiological loading. Micro-computed tomography was used to reconstruct the 3D organ-level PV geometry, which was then spatially correlated with serial en-face scanning electron microscopy imaging to quantify local collagen fiber distributions. From the acquired volume renderings, we obtained the geometric descriptors of the murine PV under increasing transvalvular pressures, which demonstrated remarkable consistency. Results to date suggest that the preferred collagen orientation was predominantly in the circumferential direction, as in larger mammalian valves. The present study represents a first step in establishing organ-level murine models for the study of heart valve disease.

## Introduction

Heart valve (HV) disease represents a significant cause of morbidity and mortality across the globe. For the diseased or congenitally abnormal semi-lunar heart valves (i.e. the aortic (AV) and pulmonary (PV) heart valves), treatment remains almost exclusively limited to replacement^1,2^. While improvements to replacement HV delivery methods have allowed for minimally invasive implantation, continued durability issues limit the longevity of this approach. This is especially true in the case for patients with congenital heart valve disease, where the PV and AV replacement is typically done at earlier ages, leading to multiple replacement surgeries^3^. Pulmonary valve malformations are responsible for a host of congenital heart malformations which are often critical and life threatening without complex surgical or interventional procedures. On the most severe end of the spectrum are in utero malformations of the pulmonary valve which cause hypoplastic right heart syndrome. Tetralogy of Fallot also frequently require right ventricular outflow tract reconstruction including placement of a valved conduit to replace the malformed pulmonary valve^4–6^. Yet, there are currently no pharmaceutical-based treatment options which can prevent or even mitigate the onset and progression of HV disease. This is in large part a result of our continued lack of understanding of the mechanisms underlying the development of valvular disease and subsequent dysfunction. Like other body systems, HV disease is thought to arise from both environmental and genetic factors^7–11^. Due to its extremely demanding biomechanical environment, HV function depends on its geometry, structure, and mechanical properties. These are in turn directly built upon the composition and organization of its extracellular matrix (ECM) and underlying cellular function^12–16^. However due to the limited or non-existence of requisite human data and continued difficulties and challenges of large animal models, our ability to place the knowledge of HV disease at the level of other cardiovascular systems remains greatly limited.

Genetically modified murine models are a well-established animal model in disease research, as the mouse shares more than 85% protein-coding genome as human^17^. Murine models provide a well-developed, relevant, and versatile animal model for investigating cellular signaling pathways. This is due to the ability to generate large number of syngenetic animals coupled with readily available techniques for manipulating the murine genome and well characterized murine genetics. Compared to large animal models, murine models are also much more cost efficient and can thus facilitate large study numbers. This makes the murine model ideal for genetic knock-out studies in the study of human HV disease.

However, a major challenge in the use of murine models in HV disease research is our ability to quantitatively characterize and simulate valvular tissue and organ level function. This is mainly due to the very small size and limited accessibility, which precludes the use of standard imaging and mechanical evaluation methods. For example, magnetic resonance imaging (MRI), conventional computed tomography (CT), and 3D echocardiography do not have the requisite spatial resolution to image the murine HV. Moreover, while quantification of the ECM components is possible using immunohistochemistry and can selectively assess target molecules, it typically only provides 2D information, omitting crucial details regarding their 3D organization. Because of the length scale spanned by HV hierarchical organization, there is no singular imaging modality that can acquire all the information necessary to describe the HV structure^15,16^. Additionally, to resolve extracellular matrix elements, high-resolution imaging techniques, such as electron microscopy (EM), are needed. However, with these techniques the field of view (FOV) is severely limited. Due to the spatial heterogeneity, isolated images captured by EM may not accurately represent fibrous structures over the entire leaflet. New methods to spatially register such very local methods with the large-scale anatomy are clearly required.

To address these issues, we developed novel methods for characterizing the functional changes in geometry and local fibrous structure of the murine heart valve under increasing transvalvular pressure across different length scales. The pulmonary valve (PV) was utilized due to its importance in congenital heart valve disease. Multiple murine PVs were prepared and fixed under physiologically relevant transvalvular pressures (TVP). We utilized a correlative imaging approach to capture key structural and geometric characteristics with increasing TVP using μCT imaging, and ECM organization with serial block face scanning electron microscopy (SBF-SEM). These novel results of this study provide the first detailed descriptors of the murine PV response to TVP at different length scales.

## Methods

### Overall study design

**Figure 1:**
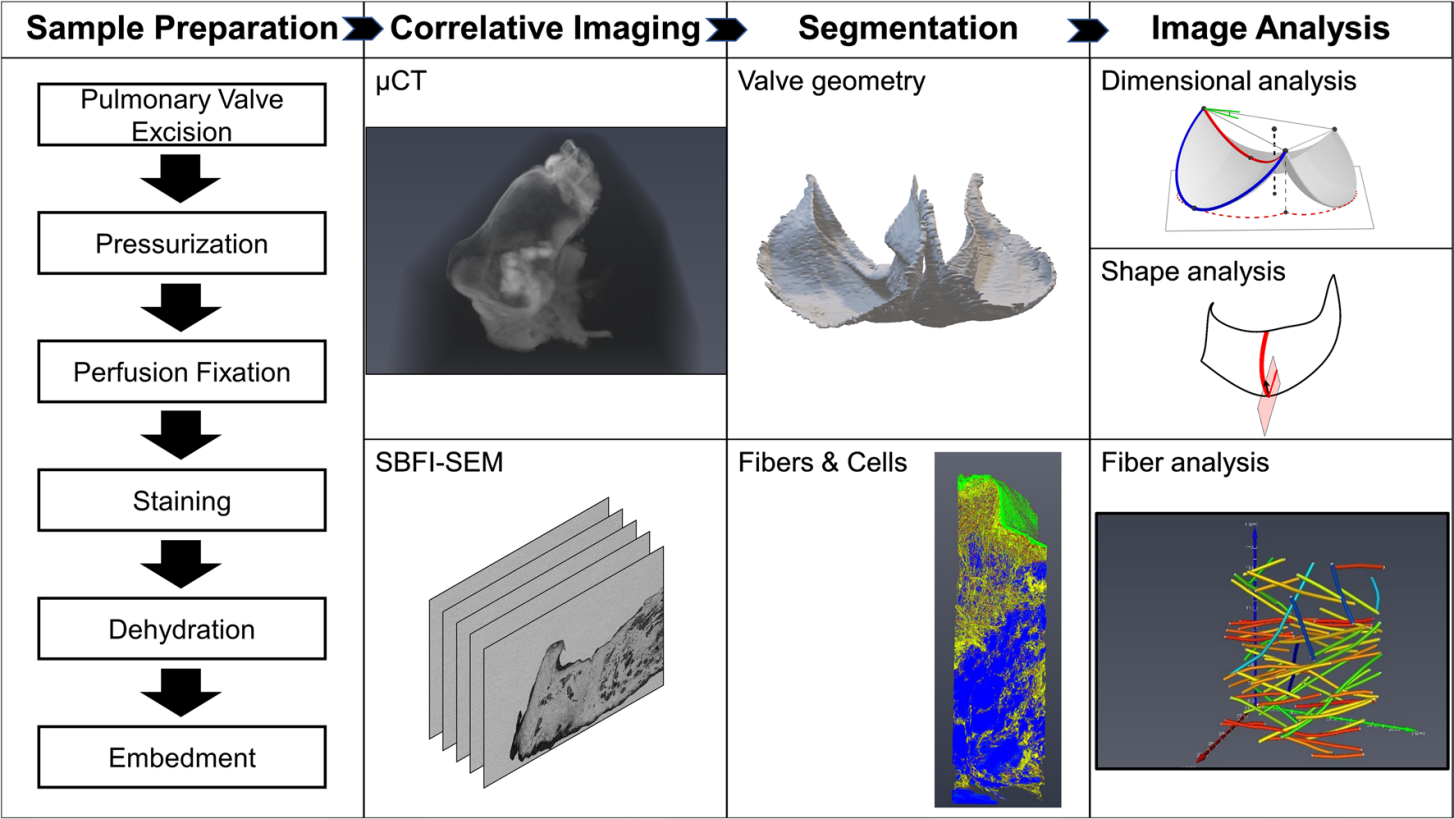
Flow chart describing the workflow for correlative imaging and analysis.

As one of our main objectives was to examine how the PV functionally adapts to increasing transvalvular pressure (TVP), our study design involved use of multiple murine PVs each fixed under a specified TVP. We utilized a comprehensive coupled experimental-modeling pipeline for each prepared PV (Fig. 1). From a total of eleven murine PVs, we developed a sufficient database to determine correlative changes with TVP. Additionally, it should be emphasized that because of the inherent heterogeneity of the PV, local fiber structure needs to be mapped back to its anatomical locations to have functional relevance. The design of the current approach allowed us to describe local fiber orientation in relation to the leaflet geometry.

### Animal preparation

All mice used in this study were approved by Nationwide Children’s Hospital institutional animal care and use committee under protocol AR13-00030. All experiments performed on mice were done in accordance with relevant guidelines and regulations of this committee. 11 C57BL/6J mice of approximately 1 year of age were sacrificed by ketamine/xylazine overdose and secondarily euthanized by bilateral thoracotomy and exsanguination. This study was done in compliance with ARRIVE guidelines^18^.

### Perfusion pressurization

To obtain organ level geometry, the PV conformation was fixed by hydrostatically applying a TVP. The heart was excised, the ventricles were removed, and the pulmonary artery (PA) was clipped approximately 2 mm above the pulmonary trunk. Polyethylene pressure monitor tubing was anastomosed to the PA with polyamide sutures. Transvalvular hydrostatic pressures were applied at 10, 20, and 30 mmHg from the arterial side with saline solution initially to ensure proper pressurization of the pulmonary trunk^19,20^. This was achieved by elevating a reservoir of solution to a height relative to the PV, which rested on the bench top. The difference in elevation between the height of the fluid versus the PV corresponded to the TVP applied from the arterial side. The reservoir was stabilized by either mounting it onto a fixed surface or by clamp, so long as the reservoir remained immobile (Supplementary Figure S1). Once confirmed, gradual incorporation of paraformaldehyde (1%), glutaraldehyde (1.25%) with CaCl_2_ (2 mM) in cacodylate buffer (0.15 M) was done over 3 h to fix the PV conformation.

### En bloc staining, dehydration, infiltration and embedding

Staining served to improve the mass-thickness contrast in µCT and EM and improve the mechanical properties of the specimen such that it was able to be sectioned by ultramicrotomy. Staining was performed using a modified reduced OTO (reduced osmium-thiocarbohydrazide-osmium) method with added uranyl acetate and lead aspartate^21–27^. Extra caution should be taken when handling osmium tetroxide and osmium reagents as it is highly toxic^28^. Dehydration was performed using successive treatments of ethanol (30%, 50%, 70%, 90%, and 100%) followed by two treatments of acetone (100%)^29^. Durcupan ACM resin (Electron Microscopy Sciences) was made according to manufacturer recommendation of 10 mL epoxy resin, 10 mL hardener, 0.3–0.4 mL accelerator, 0.1–0.2 mL dibutyl phthalate. Infiltration was done by incubating the dehydrated sample in increasing concentrations of the resin mixture in acetone: 25%, 50%, 75% each for 2 h, and 100% resin overnight. The following day, the resin mixture was replaced with a fresh 100% resin mixture and allows to sit at room temperature for 2 h. A final substitution with fresh resin was done and was cured in a 60 °C oven for 48 hours^30,31^.

### Micro-computed tomography

μCT imaging was done at the Center for Electron Microscopy and Analysis (Columbus, OH) using a Heliscan μCT (Thermo Fisher Scientific). Samples were scanned with a voxel size of 2.8 μm. Reconstruction was done in sequence with a filtered back projection algorithm. Volume rendering and image visualization was done in Avizo (Version 2020.2; Thermo Fisher Scientific) to determine locations of interest. Once a region of interest was located, the sample block was trimmed using ultramicrotomy. Using anatomical features as markers, we manually registered the virtual cross-section provided by the μCT volume rendering with the progressive cross sections of the sample via optical microscope. By comparing features on the specimen surface to the μCT cross-section, we ensure that we are along the desired slicing direction.

### Serial block face imaging scanning electron microscopy

After μCT imaging, SBF-SEM was done using a Volumescope (Thermo Fisher Scientific). The cross section of the sample block was trimmed to 2.0 × 1.5 × 1.8 mm (length × width × height) to ensure adequate slicing mechanics in the Volumescope. The sample block was coated with approximately 30 nm of gold to mitigate charging during image acquisition. Two overview regions with pixel size of 146 × 146 nm were imaged, tiled and stitched together. Higher resolution images of 10 × 10 nm pixel size were taken at local regions of interest. Approximately 1000 slices were imaged serially with thickness of 60 nm. Image segmentation, fiber detections, and fiber analysis was done in Avizo using the XFiber module.

### 3D collagen fiber registration

The µCT volume rendering served as a road map for downstream sample processing. (1) Using the virtual slices generated by the µCT rendering, the specimen block was sliced in the direction of the STJ-plane normal. (2) The physical specimen block cross-section was then corroborated with the virtual cross-section of the µCT volume rendering to verify direction and location. (3) Once the desired cross-section was exposed on the specimen block, this was then used as the initial cross-section for SBF-SEM acquisition. (4) After the SBF-SEM data set was rendered, µCT and SBF-SEM data sets were imported into Avizo and manually transformed such that they shared a common coordinate system. This allowed the fiber orientation information to be mapped to the µCT data set at a precise location on the PV.

### Segmentation of µCT images

**Figure 2:**
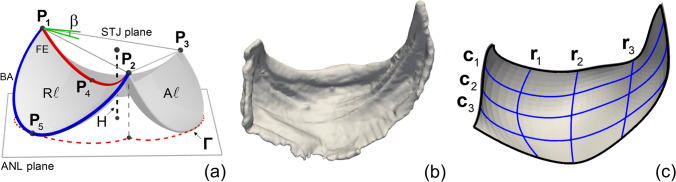
Analysis of the µCT image of individual leaflets. (**a**) schematic for the geometry of a murine PV and key geometric quantities of interest, (**b**) segmented µCT image of a single leaflet, (**c**) NURBS fitted mid-surface of the leaflet and cross sections in the circumferential and radial directions. The curves **c**_2_ and **r**_2_ represent the central cross sections of the leaflet in which **r**_2_ are chosen to pass through the mid-point of the free edge **P**_4_.

Segmentation of each valve, and as well as each constituent leaflet, from the raw µCT images was done using Synopsys’ Simpleware ScanIP (Version 2018.12-SP2; Synopsys, Inc., Mountain View, USA). The PV was first highlighted from the whole structure (including part of the myocardium, aorta, and PA). Next, the three leaflets of the PV were separated and identified as the anterior (A*ℓ*), the left (L*ℓ*), and the right (R*ℓ*) leaflets. Subsequently, we identified the commissure points as the three points (i.e., **P**_1_, **P**_2_, **P**_3_) on the interface of the segmented PV and PA at which the leaflets made contact (Fig. 2a). The basal attachment (blue curve) and free edge (red curve) of each leaflet were then defined as part of the leaflet edge between the commissure points that was or was not attached to the PA, respectively. Lastly, we defined the annulus (ANL) plane of the PV as the plane that passed through the mid-points of the basal attachments of all leaflets (e.g., **P**_5_ in Fig. 2a), and the sino-tubular junction (STJ) plane as the plane that passed through the commissure points.

### Identification and quantification of the geometric quantities of interest (gQOIs)

MATLAB (Version R2020a; Mathworks, Inc., Natick, USA), MeshLab and ParaView were used to further obtain a list of geometric quantities of interest for each segmented PV (Fig. 2c)^32,33^. These gQOIs, together with the cross-sectional shape, formed a minimal set of measurements to characterize the morphology of a PV and the constituent leaflets. From a modeling perspective, they also enabled faithful anatomical reconstruction of the PV in numerical simulations^34^. Compared to conventional echocardiography, high-resolution µCT provided more morphological details to our knowledge of gQOIs on the structure of murine PVs^35^. Particularly, for each leaflet, the free edge length L_FE_ and basal attachment length L_BA_ were measured by (1) drawing a list of nodal points on each curve with a combination of automatic edge detection and manual correction for mis-detected edge nodes, and then (2) using a 3D spline curve to fit the nodal points whose length was defined as the length of the free edge or basal attachment. The valve height, H, was computed by averaging the distance of the commissure points to the ANL plane. The leaflet thickness, w, was computed by averaging local thickness measurement at nine random locations at the belly region of the PV (three per leaflet). Finally, the valve perimeter length L_Γ_, i.e., the length of the projected contour Γ of the basal attachment of all leaflets on the ANL plane (red dotted line in Fig. 2a), was computed which we used to normalize all the above dimensional quantities (Table 1). The normalized gQOIs by the valve perimeter length were indicated by adding a bar on the non-normalized gQOIs (e.g., $\bar{L}_{{FE}}$ and $\bar{w}$). Aside from the dimensional quantities, we also measured the tilt angle β between the ANL and STJ planes.

**Table 1:** Definition and statistical analysis results for the gQOIs.

	Definition	10 mmHg	20 mmHg	30 mmHg	Intercept	Slope × 10^2^	p-value
$\overline{{\text{L}}} _{{{\text{FE}}}}$ ($\mathrm{A\ell}$)	Normalized free edge length (anterior)	0.29 ± 0.02	0.31 ± 0.01	0.30 ± 0.01	0.29 ± 0.02	0.03 ± 0.10	0.75
$\overline{{\text{L}}} _{{{\text{FE}}}}$ ($\mathrm{L\ell \& R\ell}$)	Normalized free edge length (left & right)	0.36 ± 0.01	0.35 ± 0.01	0.32 ± 0.01	0.38 ± 0.01	− 0.20 ± 0.05	0.00
$\overline{{\text{L}}} _{{{\text{BA}}}}$ ($\mathrm{A\ell}$)	Normalized basal attachment length (anterior)	0.41 ± 0.01	0.41 ± 0.01	0.41 ± 0.01	0.41 ± 0.02	0.01 ± 0.10	0.89
$\overline{{\text{L}}} _{{{\text{BA}}}}$ ($\mathrm{L\ell \& R\ell}$)	Normalized basal attachment length (left & right)	0.49 ± 0.01	0.47 ± 0.01	0.46 ± 0.01	0.49 ± 0.02	− 0.11 ± 0.09	0.21
$\overline{{\text{H}}}$	Normalized valve height	0.14 ± 0.00	0.13 ± 0.00	0.13 ± 0.00	0.15 ± 0.00	− 0.08 ± 0.02	0.01
$\overline{{\text{w}}}$× 10^2^	Normalized leaflet thickness	0.42 ± 0.04	0.36 ± 0.02	0.42 ± 0.04	0.39 ± 0.09	NA^†^	NA
β	Tilt angle (°)	11 ± 2	10 ± 2	11 ± 1	10 ± 1	NA	NA
L_Γ_	Valve perimeter length (µm)	4646 ± 20	5206 ± 67	5219 ± 206	4554 ± 257	26 ± 12^††^	0.06

The dimensional gQOIs (row 2–7) are normalized with respect to the valve perimeter length L_Γ_ (row 9), hence unitless. Column 3–5: mean ± s.e.m of the gQOIs at different TVPs. Column 6–8: mean ± s.e.m of the intercept and slope of the linear/constant regression model that fits all data at 10, 20, and 30 mmHg. The unit of the slope is mmHg^−1^ for row 2–6 and µm × mmHg^−1^ for row 9, respectively. For applicable gQOIs, the p-values were generated from the t-statistic of the hypothesis test that the slope of the linear regression model is equal to zero or not. Here,${\text{A}} \ell , {\text{L}} \ell ,{\text{R}} \ell$ indicate the anterior, left, and right leaflets, respectively.

^†^NA indicates that constant regression model is used.

^††^Actual value is reported without multiplying 10^2^ as in row 2–6.

### Leaflet shape analysis

In addition to the gQOIs, we were interested in the organ-level leaflet shape characteristics for the purposes of quantitatively understanding the functional mechanics of murine PVs under TVPs. In this study, central cross-sectional profiles of each leaflet in the circumferential and radial directions were mainly investigated. To this end, we fitted each segmented leaflet with high-fidelity using a nonuniform rational basis splines (NURBS) approach. NURBS are the gold-standard in computer aided design and manufacturing that offer flexibility and accuracy when representing complex geometries^36^. A NURBS geometry is defined by a set of NURBS basis functions and control points. For example, a generic NURBS surface **S** is defined as

1 $$ {\mathbf{S}}(\upxi ,\upeta ) = \sum\limits_{{i = 1}}^{n} {\sum\limits_{{j = 1}}^{m} {{\mathbf{R}}_{{i,j}}^{{p,q}} \left( {\upxi ,\upeta } \right)} } {\mathbf{P}}_{{i,j}} $$

in which are NURBS basis functions defined on a 2D parametric space with orders equal to *p*,*q* in the circumferential (ξ) and radial (η) directions, respectively, *n*,*m* are the number of control points along each direction, and ${\mathbf{P}}_{{i,j}}$ are the control points. Details for the construction of NURBS basis functions can be found in the Supplementary Methods.

We then fitted the segmented mid-surface of each leaflet by optimizing the position of control points from which we obtained the central cross sections of the fitted surface in the ξ and η directions (**c**_2_ and **r**_2_, Fig. 2 and Supplementary Methods). The resulting central cross sections from each PVs at the same pressure were then normalized to yield the representative shape for murine PV. Normalization scalar parameters for the circumferential and radial cross sections of each leaflet were found by projecting **c**_2_ and **r**_2_ onto the best-fitted vertical plane and determining the maximum distance in the circumferential and radial direction, respectively (Supplementary Methods). Customized codes for NURBS fitting were developed in MATLAB which used the open-source package GeoPDEs/NURBS for generation and derivatives calculation of the NURBS surface^37^.

### Statistical analysis

For each gQOI (normalized by the valve perimeter length L_Γ_), the mean and standard error of the mean (s.e.m) were calculated at each TVP (n = 3, n = 5, n = 3 for 10, 20, and 30 mmHg, respectively) (see definitions in Table 1). Additionally, linear regression analysis was conducted for each gQOI as a function of TVP. Note the exception for the normalized leaflet thickness $\bar{w}$ and tilt angle β, we applied a degenerate linear regression model with zero slope only to fit the average value for $\bar{w}$ and β. For the rest of gQOIs, the mean and s.e.m of the intercept and slope were calculated as well as the p value for the t-statistic of the hypothesis test that the slope of the linear regression model is equal to zero or not. A gQOI is considered significantly impacted by the TVP for a p-value of < 0.05. All statistical analysis was performed using the fit linear regression model function in MATLAB. Lastly, due to the observed left/right symmetry of murine PVs, we grouped the left and right leaflets together in the analysis.

## Results

**Figure 3:**
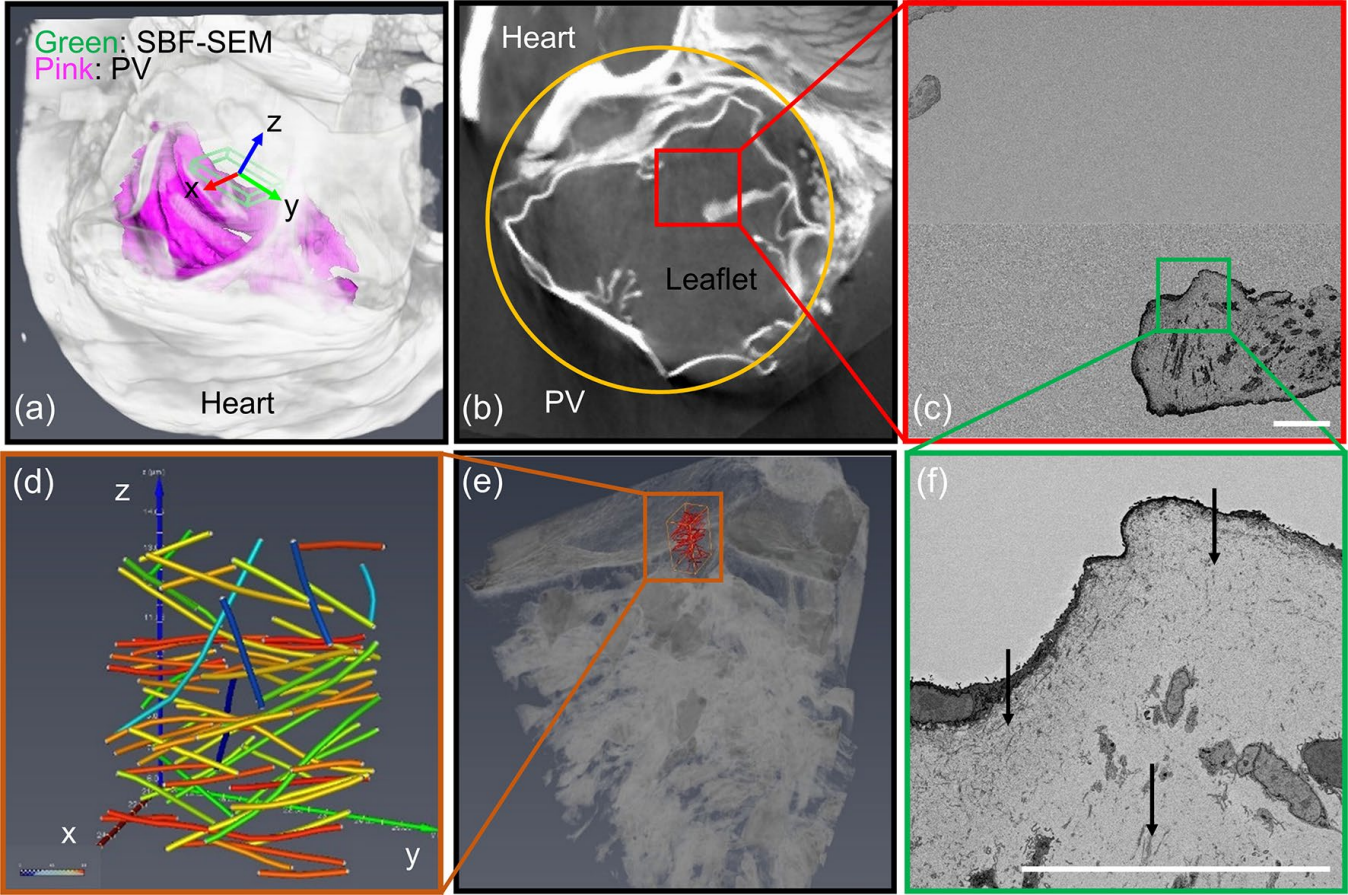
Representative images showing correlative imaging workflow. (**a**) μCT volume rendering of scanned region with a segmented PV at 20 mmHg (pink). Green bounding box (approximate dimensions of 450 × 600 × 60 μm) indicates total region imaged by SBF-SEM. (**b**) μCT volume rendering virtual slice where the PV is encircled in yellow and the red box represents the scanned area of (**c**), an image acquired by SBF-SEM. (**c**) Tiled low-resolution overview SBF-SEM micrographs of the right leaflet near the mid-point of the free edge where the green box represents the area scanned in (**f**). The contrast difference from in (**c**) indicates the two tiled and stitched micrographs. (**d**) Fiber analysis of collagen where the different colors correspond to the angular deviation from the z-axis, θ, of the SEM coordinate system which varies between 0° (blue) to 90° (red). (**e**) SBF-SEM volume rendering of detailed SEM micrographs (grayscale). Orange volume indicates subvolume where fiber (red) analysis of collagen was done. (**f**) High-resolution, detailed SBF-SEM micrograph showing ECM and cellular features. Black arrows indicate regions of collagen. Scale bars in panel (**c**,**f**) correspond to 40 μm.

The resulting μCT images and corresponding SEM images revealed high quality results, which included volume renderings containing regions of the heart, PV, and PA (Fig. 3). Multiple imaging modalities are needed because of the magnitude of length scales being traversed. The voxel size of our μCT volume renderings were between 2–5 μm, which provides sufficient resolution for gross 3D conformational information. Cellular and extracellular components require high-resolution techniques, in this case SEM, but also needed to yield architectural information. Overview images (146 × 146 nm pixel size) were taken to correlate the μCT cross-sections with SEM micrographs. Once the location was established, detailed images (10 × 10 nm pixel size) were taken to resolve extracellular matrix elements. This stepwise approach is necessary because high-resolution techniques severely limit the field of view, allowing the user to easily lose track of image location. The 2D information from individual SEM micrographs is insufficient to describe ECM architecture, thus serial images were taken and compiled to produce a high-resolution volume rendering of a localized region of the PV.

**Figure 4:**
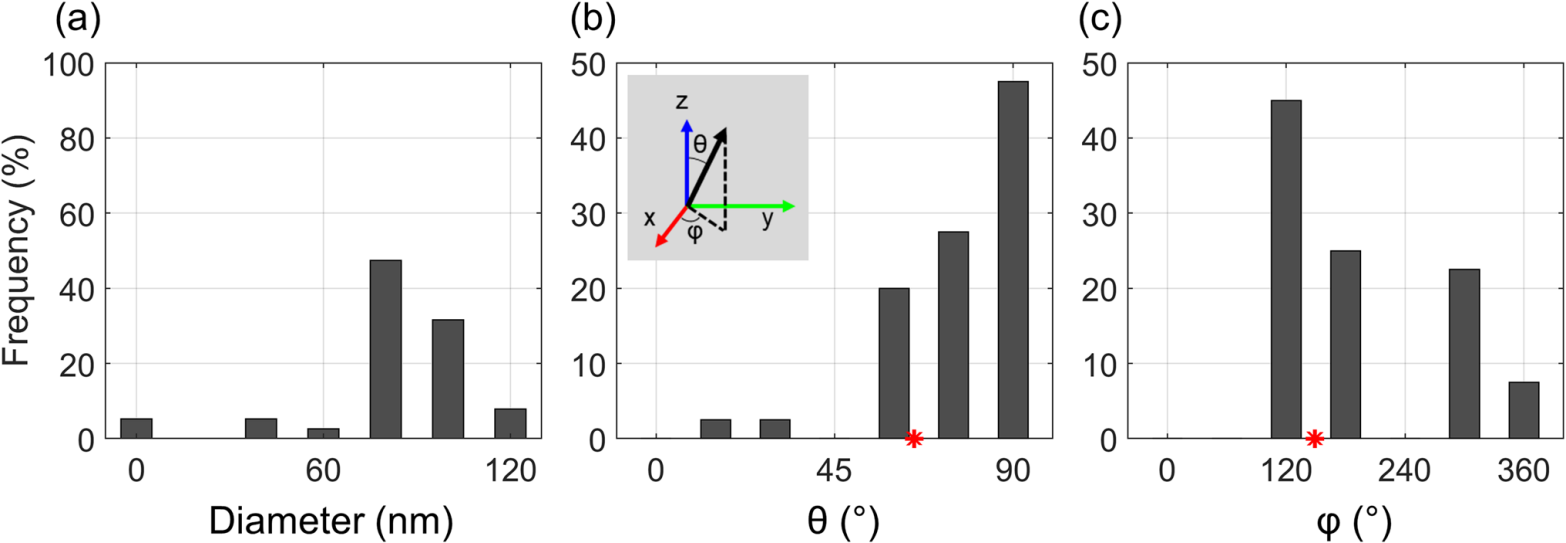
Histograms for the distribution of (**a**) diameter and (**b,c**) orientation of collagen fibers for the same location as in Fig. 3d on a right leaflet at 20 mmHg. Fibers detected using XFiber show a narrow distribution of diameters between 80–100 nm, and close alignment along the circumferential direction of the leaflet (red star). θ represents the angular deviation of a fiber with the z-axis and φ is azimuthal angle in the xy-plane with the x-axis (insert)*.* All axes refer to the coordinate system of the SEM data as is shown in Fig. 3a. The circumferential direction is drawn from the NURBS-fitted surface at the SEM location (θ = 65°, φ = 149°).

Identification and segmentation of the collagen fibers was done using the XFiber module in Avizo. In this study, we show the collagen fiber statistics for diameter and orientation in a region near the midpoint of the free edge of the leaflet (Fig. 4). The narrow peaks of the distribution indicate that collagen fiber show preferential size and direction. The orientation information, however, is according to the local coordinate system of the Volumescope and is normally arbitrary with respect to pulmonary valve anatomy. Because we used this correlative workflow, we mapped the fiber structure information to a precise location in the PV of our μCT data set (Supplementary Figure S2). The μCT and SBF-SEM data sets were manually registered to each other, aligning the coordinate systems. The rotational and lateral transformations were then applied to the fiber orientation data to determine that the collagen fibers show preferred alignment in the circumferential direction along the leaflet, consistent with literature^12,38^. It should be noted that fiber statistics was only done on a small subvolume of the PV leaflet and cannot be readily applied across the entire pulmonary valve until additional data is acquired.

**Figure 5:**
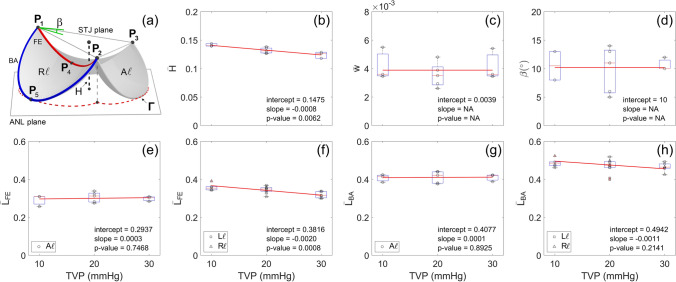
Local and global gQOIs were used to characterize the geometry of murine PVs under different TVPs. (**a**) schematic showing the key gQOIs. (**b**–**h**) show the trend of all gQOIs w.r.t TVP in which dimensional quantities were normalized using the valve perimeter length L_Γ_ (see Table 1). The boxplot with whisker displays the minimum, first quartile, median, third quartile and maximum of the data (from bottom to top) with individual measurement shown in symbols. The linear regression fit of the measurement with respect to the TVP is shown in red solid line. The p-value for the t-statistic tests the hypothesis that the slope of the regression model is equal to zero or not. As p-value is < 0.05, it indicates statistically significant change of a gQOI w.r.t. TVP.

From the µCT images, gQOIs were obtained to characterize the morphology of the PVs (Fig. 5a). The valve perimeter length, which was used to normalize the gQOIs, increased about 12% from 10 to 20 mmHg and then leveled off (Table 1). Greater variation was observed at higher TVP with the s.e.m increasing from 20 µm at 10 mmHg to 206 µm at 30 mmHg. Figure 5b–h show the trend of normalized gQOIs with respect to the TPV. It was found during analysis that the left and right leaflets were geometrically similar, while the anterior leaflet was noticeably smaller. Normalized valve height, $\bar{H}$, shows a negative dependence on the TVP (Fig. 5b). When separating the PV into individual leaflets, the normalized free edge length and basal attachment length, $\bar{L}$_FE_ and $\bar{L}$_BA_, respectively, show a similar behavior. In both instances, the anterior leaflet shows little dependence on the transvalvular pressure with average values around 0.29 and 0.41, respectively, while the left and right leaflets show a slight linear decrease in length (Fig. 5e–h). The average value for the normalized leaflet thickness $\bar{w}$ and tilt angle β is around 0.0039 (~ 19 µm + /−1 µm) and 10°, respectively (Fig. 5c,d). Note, while constant linear regression model is used in the statistical analysis, it does not mean both gQOIs do not vary with TVPs. In fact, we hypothesize that leaflet thinning occurs when the TVP increases. However, due to measurement error, we are not able to resolve this trend accurately. As a result, only average values are reported here as a reference. Mean, standard error of the mean, and linear regression parameters for the normalized free edge length and basal attachment length of each leaflet are listed in Table 1, along with the normalized valve height, leaflet thickness, and tilt angle. The statistical results suggest that the anterior leaflet is the smallest among the leaflets as characterized by a smaller normalized free edge and basal attachment lengths at all TPVs. The ratio of the mean free edge length between the anterior leaflet and left & right leaflets, which is approximately 0.81 at 10 mmHg, gradually increases to 0.94 at 30 mmHg. For the basal attachment length, similar trend is observed with the ratio increasing from 0.84 at 10 mmHg to 0.89 at 30 mmHg. These indicate the relative size of the anterior leaflet in the PV increases with increasing TVP, which is likely due to greater PA distention in the anterior direction. Note, while the normalized free edge and basal attachment lengths in the left and right leaflets decrease, there are grounds to believe that both the free edge and basal attachment are elongated when TVP increases but in a rate less than the increase of valve perimeter length. Similarly, the height of the PV is also likely to increase at larger TVP. These hypotheses, unfortunately, cannot be justified from our experiment since the absolute change of the gQOIs is not available for the same PV.

**Figure 6:**
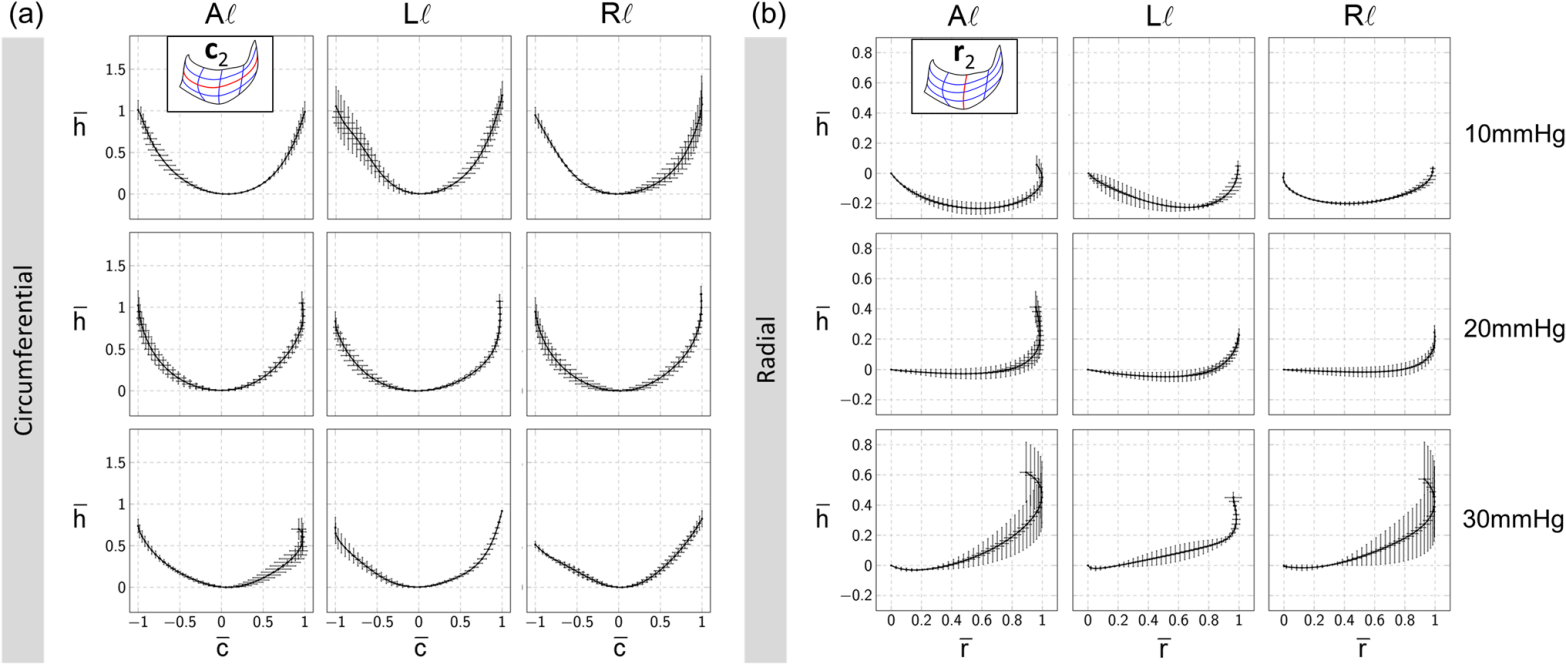
Representative normalized cross sections in the (**a**) circumferential and (**b**) radial directions, respectively. Rows/columns indicate different TVP and leaflets, respectively. Error bars were generated using the standard deviation from the cross sections of different samples at the same TVP. Each circumferential cross section is normalized such that the span in the normalized circumferential direction c̅ is from − 1 to 1, while each radial cross section is normalized to span from 0 to 1 in the normalized radial direction r̅. In (b), h̅ = 0 indicates the ANL plane, and r̅ = 0 represents the end of the radial cross section on the basal attachment. Note the consistency of the cross-sectional profiles for almost all the scenarios.

Results for shape analysis of the leaflets revealed remarkable consistency at each TVP level (Fig. 6). The plots of the individual leaflets were segregated by their overall size, where the left and right leaflets were geometrically similar and symmetrical, while the anterior leaflet was noticeably smaller. The normalized height of the leaflet, $\bar{h}$, is plotted against the outward circumferential direction (Fig. 6a), $\bar{c}$, and radial direction (Fig. 6b), $\bar{r}$, respectively, showing the cross-sectional profiles of murine PVs. By plotting these for increasing TVPs, it is more clearly revealed how the PV shape changed with TVP. Specifically, we found that in the circumferential direction, the cross sections first undergo a slightly increased inflation from 10 to 20 mmHg with little change in the overall profile. From 20 to 30 mmHg, the relative height of the cross section with respect to the span in the circumferential direction decreases noticeably for all the leaflets. In the radial direction, interestingly, that the inflation of the cross section with respect to the ANL plane is inversely proportional to the TVP. At 10 mmHg, the inflation is the most significant with almost the entire cross sections below the ANL plane for all the leaflets. At 20 mmHg and 30 mmHg, the angle of the cross section with respect to the ANL plane at the basal attachment end gradually increases leading to a lower profile of the leaflets above the ANL plane.

This phenomenon appears to be a combined effect of increasing TVP, leaflet stiffening, and distention of the PA. Qualitatively, it can be explained in the following: Increasing TVP leads to distention of the PA which stretches the leaflets in the circumferential direction. Stretched leaflets stiffens in both the circumferential and hence changes the equilibrium profile of the radial cross section. There appears to be no major difference observed in the overall leaflet profiles, except that the circumferential cross section of the anterior leaflet is approximately symmetric. Additionally, both left and right leaflets were asymmetric with respect to the circumferential direction due to the nonzero tilt angle between the STJ and ANL planes.

Overall, the normalized geometry results (Figs. 5, 6) provide a clear picture of how the murine PV adapts to TVP. Briefly, at 10 mmHg all leaflets are significantly enlarged in the radial direction. While the anterior leaflet is about 80% of the left and right leaflets in size, the profiles of the normalized central radial cross sections are similar for all the leaflets. At 20 mmHg, the anterior leaflet gets larger relatively due to the nonuniform PA distention. This contributes to a slight decrease in the normalized free edge and basal attachment lengths in the left and right leaflets. The PA distention also leads the radial cross section to become flatter at the basal attachment end with respect to the ANL plane. Meanwhile, the normalized valve height decreases slightly although the actual valve height is very likely to increase. At 30 mmHg, the above trend continues. In particular, the central radial cross section becomes even less inflated while the relative height of the central circumferential cross section noticeably decreases across all leaflets.

## Discussion

A significant hurdle that has hindered the use of murine HV has been the lack of reliable methods for determining the geometry and structure and biomechanical properties of the murine heart valve across different length scales. The size of the murine HV coupled with its heterogeneity has made it a difficult platform to study biomechanics, requiring an interdisciplinary approach. To date, most studies have been conducted either ex vivo and/or on highly aligned synthetic tissues^39–42^. Additionally, very few in literature describe the precise location of high-resolution images with respect to the heart valve location, information of critical importance when trying to describe ECM organization and, in turn, its biomechanics. A correlative workflow is necessary to describe the spatial variation within the ECM and, in turn, unify future heart valve related experiments.

Herein, we present a correlative workflow to explore the conformational changes in the murine PV as a function of TVP, using complementary imaging methods across different length scales. The novelty in this workflow lies in its ability to accurately map high-resolution micrographs to its anatomical location via μCT, while precisely controlling valve loading conditions. In doing so, we are able to drastically reduce the effects of temporal and spatial heterogeneity in experimentation. We were then able to provide the first quantifiable geometric parameters and shape characteristics of the murine PV that can be used to determine its biomechanical properties. The narrow variance of the derived geometric parameters was indicative of remarkable experimental reproducibility, and inherently addresses sample variation. This is further reinforced by the capability of resolving and quantifying collagen distributions with serial imaging scanning electron microscopy.

Yet, the present study is only a first step; a complete understanding of the etiology of HV disease remains an elusive target. Given its primary mechanical function, the geometric and biomechanical properties of an HV clearly determine its functionality. These properties arise as a direct result from the composition and structure of the valvular ECM, which in turn is produced and remodeled by the valvular interstitial cells which make up the valve tissue. Therefore, elucidating the mechanisms underlying growth and remodeling of the valvular ECM holds the key to understanding its physiology and pathophysiology. Multiple transgenic murine models exist which can be used to investigate the growth and remodeling of the ECM in the murine heart valve^43–47^. For example, certain osteogenesis imperfecta murine model with aortic valve disease phenotype is known to present noticeable ECM remodeling in the valve^48^. While it has been shown that such remodeling is associated with excessive proteoglycan deposition and cell proliferation, little connection has been made between morphological and cellular change of the valve and organ-level valvular function. Even less is known about the structural development of ECM over time.

The workflow and methodology described is not without their limitations. Due to the acquisition times associated with high-resolution imaging, we only acquired and analyzed one SBF-SEM data set. While it is sufficient to demonstrate the pipeline for this study, further investigation is necessary to image the ECM in its entirety and to understand collagen fiber distribution of murine PVs. As a reference, the high-resolution SBF-SEM work presented here required approximately one week of continuous imaging. Instead, it is more feasible to strategically sample specific areas of the PV to obtain a representation of ECM organization. The next step in this workflow development would be to determine which areas need to be sampled and how many replicates would be needed. In addition, our current workflow limits us to studying a single valve fixed at a single pressure which is inefficient. Future iterations of this correlative workflow can come in the form of in operando pressurization of the PV. Though this work has shown promising results, it is desirable to pressurize the same heart at different pressures to eliminate any sample-to-sample variation. This would involve engineering an enclosed pressure chamber where the heart would remain hydrated and maintain a transvalvular pressure for the duration of a μCT scan. The challenges of this would be (a) ensuring that the pressurization tubing would not interfere for the path of the X-rays; (b) creating a system where sample movement is less than 10 voxels to ensure adequate reconstruction; and (c) honing scan parameters for low contrast imaging since the sample can no longer be chemically fixed and stained for repeatable pressurizations.

Though this work shown focus exclusively on the PV, the correlative workflow can be applied to any murine tissue to reveal its hierarchical organization. Once a correlative workflow is established, this can readily integrate complementary components. Characterization of molecular mechanisms, flow conditions, and transgenics can be integrated to build an indiscriminate platform for experimentation. Additional imaging techniques can also be appended to the workflow depending on the data desired. The relationship of strain states to collagen fidelity has been understudied and needs high-resolution imaging instruments in transmission electron microscopy or scanning transmission electron microscopy^49,50^. Developing applications in cryo-EM will potentially allow the use of local chemical analyses to determine local variations in collagen^51^. Related valve geometry data acquired by echocardiography can provide in vivo hemodynamic conditions of the murine PV, once spatial and temporal resolution issues are resolved^52^. We thus present this work with the intention of providing a foundation and precedence for future valve experimentation.

Lastly, it is to be noted that, with further numerical simulation and inverse modeling, the gQOIs and valvular cross-sectional profiles that we consistently obtained across TVPs allow for characterization of the nonlinear mechanical properties of murine PVs. This, though, is beyond the scope of this study.

## Conclusions

In this work, we present a correlative workflow to control the conformation of the PV and using different imaging modalities to traverse the breadth of length scales needed to describe the PV architecture. In doing so, we provide the first quantifiable geometric parameters and shape characteristics of the murine PV.

## Supplementary Information

Supplementary Information.
