# Supplementary material for: On the shape and structure of the murine pulmonary heart valve
Source: Sci Rep. 2021 Jul 7;11:14078. doi: 10.1038/s41598-021-93513-0 (PMC8263753; doi:10.1038/s41598-021-93513-0)
Supplement: Supplementary file 1 — Supplementary Information. [file 41598_2021_93513_MOESM1_ESM.pdf]

ON THE SHAPE AND STRUCTURE OF THE MURINE PULMONARY HEART VALVE

Yifei Liu[1,2], Xinzeng Feng[3], Hao Liu[3,4], David W. McComb[1,2],
Christopher K. Breuer[5,6], and Michael S. Sacks[3,4*]

[1] Center for Electron Microscopy and Analysis
The Ohio State University, Columbus, OH 43210, USA
[2] Department of Materials Science and Engineering
The Ohio State University, Columbus, OH 43210, USA

[3] Willerson Center, Oden Institute for Computational Engineering and Sciences
The University of Texas at Austin, Austin, TX 78712, USA
[4] Department of Biomedical Engineering
The University of Texas at Austin, Austin, TX 78712, USA

[5] Center for Regenerative Medicine, Abigail Wexner Research Institute
Nationwide Children's Hospital, Columbus, OH 43205, USA
[6] Department of Pediatric Surgery
Nationwide Children's Hospital, Columbus, OH 43205, USA

[*] Correspondence to msacks@oden.utexas.edu.

SUPPLEMENTARY FIGURES

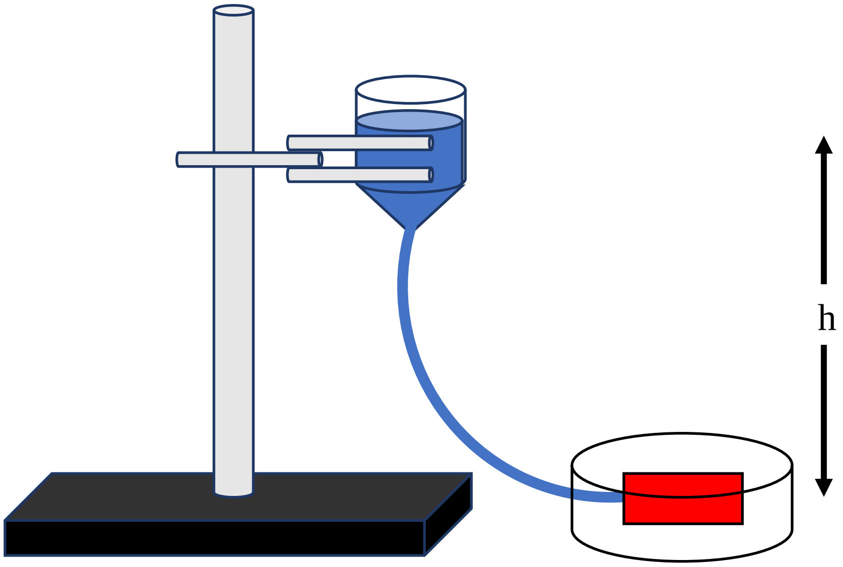

Figure S1. A schematic illustrating the process of perfusion pressurization. By varying the fluid height, h, relative to the excised PV (red container), the applied TVP is changed.

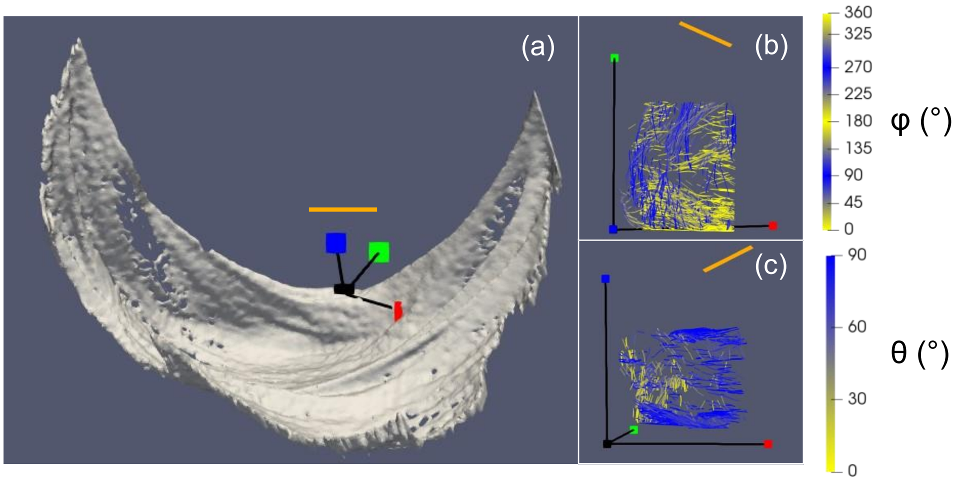

Figure S2. (**a**) Local coordinate system of SBF-SEM imaging overlayed on 3D volume rendering of the segmented µCT data. Red, green, and blue points indicate the x, y, and z axes, respectively. (**b-c**) Different views of collagen fibers color coded using the solid angles φ and θ, respectively. The orange line indicates the circumferential direction of the leaflet at the location of SBF-SEM imaging.

SUPPLEMENTARY METHODS

NURBS fitting procedure. For each leaflet, the fitting NURBS surface **S** is defined on the unit square Δ = [0,1]×[0,1] with 7 control points in the circumferential (ξ) direction and 4 control points in the radial (η) direction (Fig. S3). In both directions, **S** is clamped at the ends and 4th-order NURBS basis functions are used, i.e., $n = 7$, $m = 4$, $p = q = 4$ in equation (1). Additionally, equal weights and uniform knot vectors (i.e., the difference between any two consecutive knots remains fixed) are assumed. The position of the control points is determined by optimizing the fitness of the NURBS surface to the raw segmented leaflet volume. To compute the fitness for a given set of control points, we first generate a set of points $\mathcal{P}=\{\mathbf{p}_{\mathrm{k}}\}$ on the NURBS surface **S** by evaluating equation (1) on a uniform 40×40 grid in Δ. The point set $\mathcal{P}$ is then compared against another point set $\mathcal{Q}=\{\mathbf{q}_{\mathrm{n}}\}$ which contains the points randomly downsampled from the total vertices of the leaflet. The cost function for comparing $\mathcal{P}$ and $\mathcal{Q}$ is defined by:

$$D^*(\mathcal{P},\mathcal{Q}\,|\,\mathbf{P}_{\mathrm{ij}}) = \frac{1}{|\mathcal{P}|}\sum_{\mathrm{k}}\left\|\mathbf{p}_{\mathrm{k}}-\mathbf{q}_{\mathrm{k}}^*\right\| + \frac{1}{|\mathcal{Q}|}\sum_{\ell}\left\|\mathbf{q}_{\ell}-\mathbf{p}_{\ell}^*\right\| + \frac{\alpha}{\iint J\,\mathrm{d}\xi\mathrm{d}\eta}\iint\left(\left\|\frac{\partial^2\mathbf{S}}{\partial\xi\partial\xi}\right\|^2 + \left|\left\|\frac{\partial^2\mathbf{S}}{\partial\xi\partial\eta}\right\|^2 + \left|\left\|\frac{\partial^2\mathbf{S}}{\partial\eta\partial\eta}\right\|^2\right) J\,\mathrm{d}\xi\mathrm{d}\eta \qquad \text{(S1)}$$

where $\mathbf{q}_{\mathrm{k}}^* \in \mathcal{Q}$, $\mathbf{p}_{\ell}^* \in \mathcal{P}$ denote the nearest points to the point $\mathbf{p}_{\mathrm{k}}$ and $\mathbf{q}_{\ell}$ in $\mathcal{Q}$ and $\mathcal{P}$, respectively, $\|\bullet\|$ represents the Euclidean norm in space, α is a scalar coefficient of Soblev regularization introduced to improve the smoothness of the resultant NURBS surface, and

$$J = \left\|\frac{\partial\mathbf{S}}{\partial\xi}\times\frac{\partial\mathbf{S}}{\partial\eta}\right\| \qquad \text{(S2)}$$

is the Jacobian of NURBS representation. Numerically, we find $|\mathcal{Q}| = 2000$ leads to good balance between accuracy and efficiency and $\alpha = 5\times10^{-6}$ is used in practice[53].

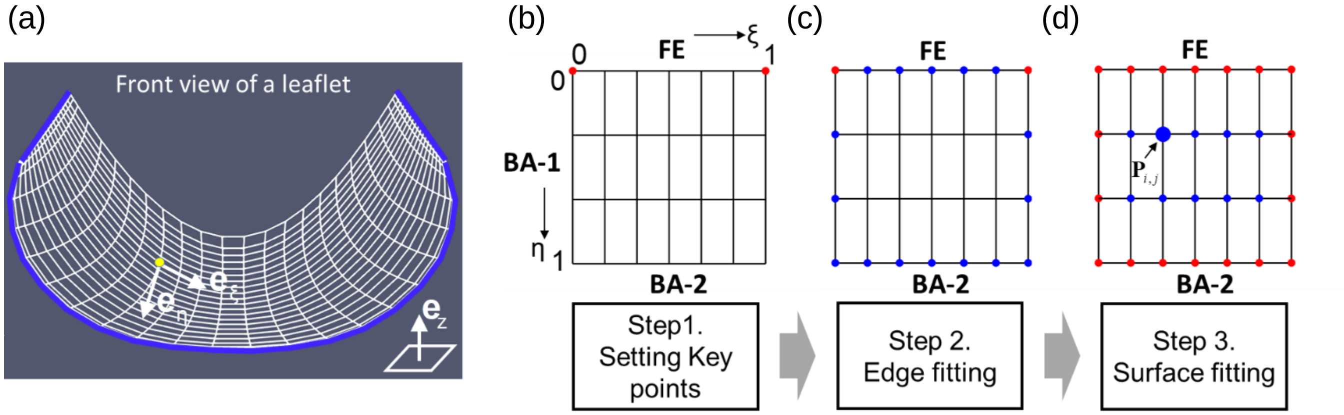

Figure S3. A schematic illustrating the steps for NURBS surface fitting of a leaflet. (**a**): Front view of the NURBS surface for a leaflet. $\mathbf{e}_\xi$, $\mathbf{e}_\eta$ indicate the local circumferential and radial directions of the surface and $\mathbf{e}_z$ indicates the normal direction to the ANL plane. (**b-d**): steps for NURBS surface fitting. The unit square is the parametric domain Δ on which the NURBS surface is defined. The 4x7 grid indicates the topological arrangement of control points ($\mathbf{P}_{1,1}$ located at the upper left corner). Those along the ξ axis are mapped to the circumferential direction of the leaflet while those along the η axis are mapped to the radial direction. In each step, the control points in red or blue are either fixed or optimized. (**b**): in step 1, the positions of the two commissure points are set manually. (**c**): in step 2, the rest of the control points on the leaflet edge are optimized. (**d**): in step 3, the interior control points are optimized.

The above optimization problem yields around 80 unknowns. To improve convergence of the solver, fitting is performed in three steps (Fig. S3). In the first step, the positions of the two commissure points are manually determined which the control points $\mathbf{P}_{1,1}$ (top left corner), $\mathbf{P}_{7,1}$ (top right corner) are set to. In the second step, the rest of the control points on the edge of the parametric domain are optimized to fit the free edge and basal attachment of the leaflet, respectively. In this step, the cost function (Equation S1) is used but without the regularization term. In the third step, with all the control points on the edge fixed, the interior control points are optimized to minimize D^*. Note, since the point set $\mathcal{Q}$ is drawn from the total vertices of the segmented leaflet and hence randomly distributed on the upper and bottom surfaces, the fitted NURBS surface **S** shall be considered as a smooth approximation of the leaflet mid-surface. Numerically, all optimization is performed in MATLAB using the open-source package GeoPDEs/NURBS for generation and derivatives calculation of the NURBS surface.

Notes for general NURBS construction. Once the weight associated with each control point and the non-decreasing knot vectors in both directions are specified, one can construct the NURBS basis functions following: 1) generate the univariate B-spline basis functions using the Cox-de Boor recursive relations in each direction, 2) rationalize the weighted univariate B-spline basis functions, and then 3) form the bi-variate NURBS basis functions using tensor product[54]. For simplicity, uniform knots vectors and weights are assumed in this manuscript.

Processing of cross sections. Here, our goal is to develop the representative cross-sectional profiles for different leaflets and TVP. This is done by 1) postprocessing the NURBS fitted surface to obtain characteristic cross sections for each PV, and 2) "averaging" the cross sections obtained from different

PVs at the same TVP to develop the representative cross-sectional profiles. Particularly, we are interested in the central cross sections in the circumferential and radial directions of each leaflet (see Fig. 2) which characterize the main shape of the PV. The details for postprocessing the NURBS fitted surface and averaging the cross sections are described in the following.

In the first step (Fig. S4a), we draw the "initial" central circumferential and radial cross sections from the NURBS fitted surface $\mathbf{S}$ which correspond to $\eta = 0.5$ and $\xi = 0.5$ in the NURBS parametric domain Δ, respectively. Due to sample distortion, we noticed that the central radial cross sections drawn in this way may not always lie in the center of the leaflet. Therefore, slight manual correction is introduced such that the corrected radial cross section passes through the mid-point of the free edge. The corrected

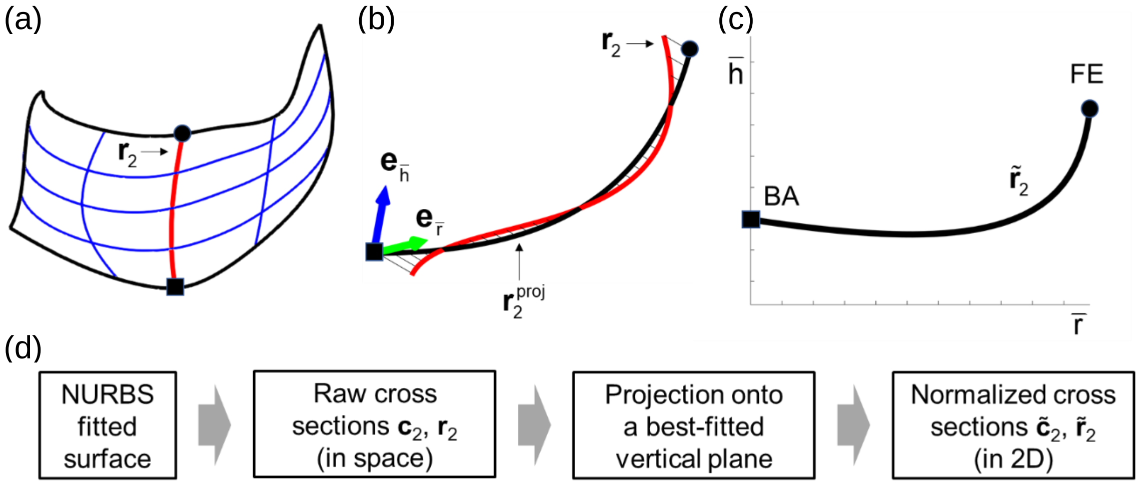

Figure S4. Schematics illustrating the processing of normalized cross sections in Fig. 6. For clarity, we use radial cross section as an example. (**a**): Raw central radial cross section $\mathbf{r}_2$ (red highlighted) on a NURBS fitted surface. The solid black circle and square indicate the end points of the cross section on the free edge (FE) and basal attachment (BA), respectively. (**b**): The raw cross section (red) is projected onto a best-fitted plane (vertical to the ANL plane), yielding $\mathbf{r}_2^{proj}$ (black). The coordinate axes of the plane in the height and radial directions are denoted as $\mathbf{e}_{\bar{h}}, \mathbf{e}_{\bar{r}}$, respectively. Thin lines between $\mathbf{r}_2$ and $\mathbf{r}_2^{proj}$ connect the original point to the projected point on the vertical plane. (**c**): Finally, the projected radial cross section is normalized such that the span of the normalized radial cross section equals to 1 along $\mathbf{e}_{\bar{r}}$, yielding $\tilde{\mathbf{r}}_2$. (**d**): flow chart for the entire process.

central radial cross section is defined by $\xi = \xi_0$ on the NURBS surface where $\xi_0 \in [0.4,0.6]$ varies from sample to sample. No correction is applied on the circumferential cross section. In the following, we shall call the corrected cross sections as the raw central cross sections in the circumferential and radial directions, respectively.

In the second step (Fig. S4b), we project the raw central cross sections onto a vertical plane to the ANL plane. While the out-of-plane component of the raw cross sections is discarded during the projection, we argue that it is a high-order component sensitive to data distortion and numerical error. On the vertical plane, natural coordinate system is introduced with one unit coordinate axis $\mathbf{e}_{\bar{h}}$ along the height direction and the other $\mathbf{e}_{\bar{c}} = \mathbf{e}_{\bar{h}} \times \mathbf{N}$ for the circumferential cross sections, and $\mathbf{e}_{\bar{r}} = \mathbf{e}_{\bar{h}} \times \mathbf{N}$ (pointing

from the basal attachment to the free edge) for the radial cross sections. Here, $\mathbf{N}$ is the unit normal vector of the vertical plane. Denote the projected circumferential and radial cross sections as

$$c_2^{proj} = \left\{\left(x_{c,i}, y_{c,i}\right)\right\}_{i=1}^{K_c}, r_2^{proj} = \left\{\left(x_{r,i}, y_{r,i}\right)\right\}_{i=1}^{K_r} \quad \text{(S3)}$$

, respectively, in the local $\mathbf{e}_{\bar{c}}$ - $\mathbf{e}_{\bar{h}}$ or $\mathbf{e}_{\bar{r}}$ - $\mathbf{e}_{\bar{h}}$ coordinate systems. Here, K_c and K_r is the number of mark points on each curve. Without losing generality, we also enforce the constraint that the mark points on both curves are equally spaced.

In the third step (Fig. S4c), we normalize the projected central circumferential and radial cross sections. Specifically, let X, Y denote the span of the projected circumferential or radial cross sections along $\mathbf{e}_{\bar{c}}$ and $\mathbf{e}_{\bar{r}}$, i.e., $X = \max_i(x_{c,i}) - \min_i(x_{c,i})$, $Y = \max_j(x_{r,j}) - \min_j(x_{r,j})$. Then, the normalized circumferential and radial cross sections are defined as

$$\tilde{\mathbf{c}}_2 = \left\{\left(2x_{c,i} / X + \delta_{cx}, 2y_{c,i} / X + \delta_{cy}\right)\right\}_{i=1}^{K_c}, \tilde{\mathbf{r}}_2 = \left\{\left(x_{r,i} / Y + \delta_{rx}, y_{r,i} / Y\right)\right\}_{i=1}^{K_r} \quad \text{(S4)}$$

in which δ_{cx}, δ_{rx} are chosen such that the normalized circumferential and radial cross sections span from -1 to 1 or from 0 to 1, respectively, and $\delta_{cy} = -2\min_i(y_{c,i})/X$ translates the circumferential cross sections to the same reference height at the lowest point.

After above steps of postprocessing, the normalized cross sections from different PVs are compiled and used to compute the representative cross sections for different leaflets and TVPs (see Fig. 6). To construct the representative cross sections, we take the average of the position of corresponding mark points on each normalized curve. For example, denote the normalized central circumferential cross sections for all 5 anterior leaflets at 20mmHg to be $\tilde{\mathbf{c}}_2^{A1}, \tilde{\mathbf{c}}_2^{A2}, \tilde{\mathbf{c}}_2^{A3}, \tilde{\mathbf{c}}_2^{A4}, \tilde{\mathbf{c}}_2^{A5}$. Then the representative central circumferential cross section for the anterior leaflet at 20mmHg is

$$\tilde{\mathbf{c}}_2^{A} = \left\{\left(\frac{1}{5}\left(x_{c,i}^{A1} + x_{c,i}^{A2} + x_{c,i}^{A3} + x_{c,i}^{A4} + x_{c,i}^{A5}\right), \frac{1}{5}\left(y_{c,i}^{A1} + y_{c,i}^{A2} + y_{c,i}^{A3} + y_{c,i}^{A4} + y_{c,i}^{A5}\right)\right)\right\}_{i=1}^{K_c}. \quad \text{(S5)}$$

Finally, the standard deviation at different mark points is used to generate the error bars in Fig. 6. Numerically, all postprocessing is done with customized codes in MATLAB.
